# Construction and comprehensive analysis of the competing endogenous RNA network in endometrial adenocarcinoma

**DOI:** 10.1186/s12863-022-01028-y

**Published:** 2022-02-06

**Authors:** Chong Feng, Lei Cui, Zhen Jin, Lei Sun, Xiaoyan Wang, Xinshu Chi, Qian Sun, Siyu Lian

**Affiliations:** 1 Shengjing hospital affiliated to China medical university, No.36 ,Sanhao street, Heping district, Shenyang, Liaoning province China; 2 School of health management, China medical university, No. 77, Puhe road, Shenbei new district, Shenyang, Liaoning province China

**Keywords:** Endometrial carcinoma, Competing endogenous RNAs co-expression, Weighted gene co-expression network analysis, Hub gene

## Abstract

**Background:**

Endometrial carcinoma (EC) is one of the most common gynecological malignant tumors. In this study, we constructed gene co-expression networks to identify key modules and hub genes involved in the pathogenesis of EC.

**Results:**

The MEturquoise module was found to be significantly related to hypertension and the MEbrown module was significantly related to the history of other malignancies. Functional enrichment analysis showed that the MEturquoise module was associated with the GO biological process terms of transcription from RNA polymerase II promoter, positive regulation of male gonad development, endocardial cushion development, and endothelial cell differentiation. The MEbrown module was associated with GO terms DNA binding, epithelial-to-mesenchymal transition, and transcription from RNA polymerase II promoter. A total of 10 hub genes were identified and compared with the available datasets at transcriptional and translational levels.

**Conclusions:**

The identified ceRNAs may play a critical role in the progression and metastasis of EC and are thus candidate therapeutic targets and potential prognostic biomarkers. The two modules constructed further provide a useful reference that will advance understanding of the mechanisms of tumorigenesis in EC.

**Supplementary Information:**

The online version contains supplementary material available at 10.1186/s12863-022-01028-y.

## Background

Endometrial carcinoma (EC) is one of the most common gynecological tumors worldwide, and its incidence has been on the rise every year in both developed and developing countries [1]. Kessler et al. [2] reported that the incidence of EC remained unchanged in women aged 50–74 years from 1992 to 2002, but increased by 2.5% annually from 2006 to 2012. This significant increase in the incidence of EC in recent years is attributed to a change of lifestyles and dietary structure, along with informal hormone replacement therapy and abuse of sex hormones; however, the use of vaginal estrogen was found to not increase the incidence of EC [3]. Moreover, patients diagnosed with EC are becoming younger, representing a serious threat to women’s health [4, 5]. In 2014, The World Health Organization classified EC according to histological type as endometrioid, serous, mucinous, clear cell, neuroendocrine, mixed, undifferentiated/dedifferentiated, and others. Among these types, adenocarcinoma, clear cell carcinoma, serous papillary adenocarcinoma, squamous cell carcinoma, and undifferentiated carcinoma are highly malignant, whereas squamous cell carcinoma and undifferentiated carcinoma are relatively rare. EC has a known hereditary component. In patients without metastatic tumors, the 5-year survival rates range from 74 to 91%. However, there is a lack of statistical data based on appropriate methods for large-scale screening of EC in an asymptomatic population, and there is no relevant guideline for screening of EC in the asymptomatic general population or medium-risk population. In contrast to other gynecological tumors such as ovarian cancer and cervical cancer that are associated with highly specific landmark molecules such as CA125, HE4, and SCC [6–8], no such markers are available for EC, which is a challenge for clinical diagnosis. The diagnosis of EC thus mainly depends on imaging, immunohistochemistry, pathology, and hysteroscopy [9–12]. At present, it is considered that transvaginal ultrasound measurement of endometrial thickness [9, 13, 14] and endometrial aspiration cytological examination are feasible technologies for EC screening. Dilatation and curettage [15, 16] and hysteroscopy are also potentially effective screening methods, but are highly invasive and therefore not recommended. Therefore, further effort is needed to identify candidate molecular markers to improve the screening and early diagnosis of EC, as well as to provide insight into the pathogenic mechanism.

Salmena et al. [17] proposed the competing endogenous RNA (ceRNA) hypothesis in 2011, suggesting that mRNAs, transcribed pseudogenes, and long non-coding RNAs (lncRNAs) form a ceRNA network through microRNA (miRNA) response elements (MREs), which plays an important role in tumor formation. The traditional view of gene regulation is that an miRNA binds to its target mRNA to affect the translation and stability of the target gene. This binding of the miRNA at the MRE can induce the target gene mRNA to degrade or inhibit its translation into a protein to regulate gene expression at the post-transcriptional level [18, 19]. Seitz et al. [20] confirmed that transcribed pseudogenes and lncRNAs could be competitively bound to miRNA at the binding sites, resulting in down-regulation of the activity and quantity of the miRNA, which would remove the inhibitory effect on the downstream target mRNA. Based on this background, we aimed to identify potential markers of EC by identifying differentially expressed RNAs in endometrial adenocarcinoma and non-cancer tissues, which were used to construct a ceRNA network.

In this study, we constructed gene co-expression networks for endometrial carcinoma (EC) according to the competing endogenous RNAs (lncRNAs, mRNAs, miRNAs) identified to be differentially expressed between normal and adenocarcinoma tissues from patient samples included in The Cancer Genome Atlas database (TCGA), a joint project supervised by the National Cancer Institute and the National Human Genome Research Institute, which aims to use high-throughput genomic analysis techniques to facilitate research toward gaining a better understanding of cancer, and ultimately improve the ability to prevent, diagnose, and treat cancer. Weighted gene co-expression network analysis was used to identify modules containing the various differentially expressed RNAs and their predicted or known target genes, and the hub genes of the networks were subjected to functional annotation. Overall, we identified two modules containing various independent genes, which were related to hypertension and history of other malignancies, respectively. The comprising genes were significantly enriched in Gene Ontology (GO) terms and Kyoto Encyclopedia of Genes and Genomes (KEGG) pathways that are likely linked to tumorigenesis.

Our study makes a significant contribution to the literature because the incidence of EC has been increasing in recent years, with an alarming increase in younger patients. However, there is still no molecular marker for early detection and prognosis prediction, and the detailed pathogenic mechanisms remain unclear. Therefore, the identified hub genes and modules from this study offer a valuable reference and starting point for investigating biomarkers and potentially new drug targets, along with providing insight into the mechanisms of tumorigenesis of EC.

## Methods

### Data collection

The transcripts of the TCGA-UCEC (disease type: endometrial adenocarcinoma) database were downloaded, comprising count and miRNA-Seq data expressed by RNAs. The downloaded data were preprocessed to form a gene expression matrix, the mRNAs and lncRNAs in the count data matrix were separated, and the miRNA matrix was added to obtain three separate matrices for co-expression analyses.

### Differential gene expression analysis

We used R 3.5.1 software with the R packages edger, gplots, and pheatmap to extract differentially expressed genes (DEGs) from the dataset and draw volcanic and thermal maps. The criteria for DEG identification were an absolute log fold-change value > 2 and false discovery rate (adjusted *p*-value) < 0.01. The differentially expressed lncRNAs and miRNAs were then compared in the miRcode database to screen out the lncRNA-miRNA relationships specific to endometrial adenocarcinoma. After the compared miRNAs were modified for 3p,5p standardization in the starBase database, their target genes were predicted using the miRDB, miRTarBase, and TargetScan tools, and then the miRNA-mRNA relationships of endometrial adenocarcinoma were obtained. The predicted target genes were intersected with the screened differentially expressed RNAs, and a Venn diagram was obtained using the venndiagram R package. After comparison with mRNAs, the miRNAs were compared with the selected differentially expressed lncRNAs to identify related lncRNA-miRNA pairs. Finally, the ceRNA network diagram of differentially expressed RNAs in EC was constructed with Cytoscape.

### WGCNA

WGCNA was performed based on the constructed ceRNA network and patient characteristics, including hypertension, diabetes, history of colorectal cancer, history of other malignancies, and new tumor events. Randomly selected cancer samples from the TCGA database were clustered and the abnormal samples were removed according to a certain height. After loading the trait data, the removed samples were regrouped and linked to the trait data to make a heat map. A relationship matrix of the expression data was constructed, followed by a topology matrix according to the fitting indices (R^2^ and mean connectivity with a soft threshold (power) value), and the final scale-free network. According to tom-based dissimilarity, the average method was used to cluster the genes, and the dynamic shearing tree method was used to identify the modules.

### GO and KEGG enrichment analysis

The Database for Annotation, Visualization and Integrated Discovery (DAVID) v. 6.8 tool comprises a full Knowledgebase update to the sixth version of the original web-accessible programs. DAVID now provides a comprehensive set of functional annotation tools to interpret the biological meaning behind large lists of genes (https://david.ncifcrf.gov/home.jsp). KOBAS 3.0 is a web server for gene/protein functional annotation (Annotate module) and functional gene set enrichment (http://kobas.cbi.pku.edu.cn/). Genes from two of the identified modules showing significant associations with patient characteristics, MEbrown and Meturquiose, were respectively input in the DAVID database for functional annotation and enrichment from the GO biological process, cellular component, and molecular function terms, followed by KEGG pathway enrichment.

### Selection and verification of hub genes

Highly connected nodes in a network are considered to play an important role in the stability of the network. Therefore, in the MEbrown and MEturquiose modules, the genes with nodal degree > 3 were selected as candidate hub genes. These hub genes were analyzed with respect to their relationship to patient survival in GEPIA (http://gepia.cancer-pku.cn/), a newly developed interactive web server for analyzing RNA-seq expression data of 9736 tumors and 8587 normal samples from the TCGA and GTEx projects using a standard processing pipeline. In this study, genes with a log rank value < 0.05 were selected from the two modules as potential hub genes in the endometrial adenocarcinoma genetic network. The transcriptional levels of the hub genes were verified in The Human Protein Atlas database (https://www.proteinatlas.org/), a Swedish-based program initiated in 2003 with the aim to map all of the human proteins in cells, tissues, and organs using integration of various omics technologies, including antibody-based imaging, mass spectrometry-based proteomics, transcriptomics, and systems biology.

### Genetic alteration of hub genes

The genomic changes in candidate hub genes and their correlations were assessed with cBioPortal for Cancer Genomics, which provides tools for the visualization, analysis, and download of large-scale cancer genomics datasets. Complex cancer genomic profiles can be easily obtained using the query interface of the portal, enabling researchers to explore and compare genetic alterations across samples, and to search for potential targets of anti-tumor drugs.

## Results

### Data download

RNA-seq data of endometrial adenocarcinoma were downloaded from the TCGA dataset, comprising a total of 425 samples, including 19 normal samples and 406 cancer samples. The miRNA-seq data comprised a total of 423 samples, including 18 normal samples and 405 cancer samples.

### DEG identification

**Fig. 1:**
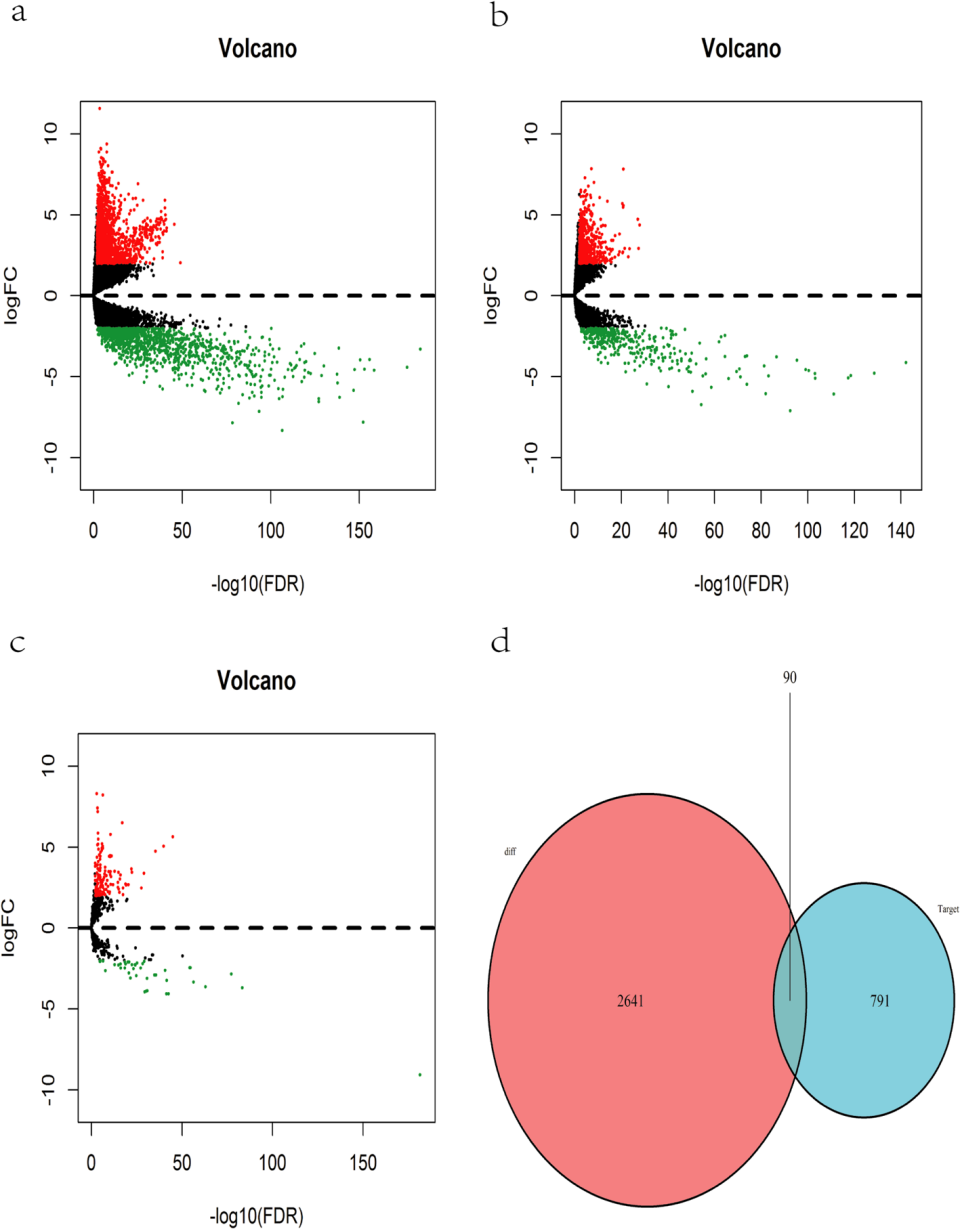
Volcano plot and Venn diagram of differentially expressed RNAs in endometrial carcinoma (EC). **a** Volcano plot visualizing differentially expressed genes (DEGs) at the mRNA level. **b** Volcano plot visualizing DEGs at the lncRNA level. **c** Volcano plot visualizing DEGs at the miRNA level. **d** Venn diagram representing the intersection of predicted target genes and the screened differentially expressed RNAs

**Fig. 2:**
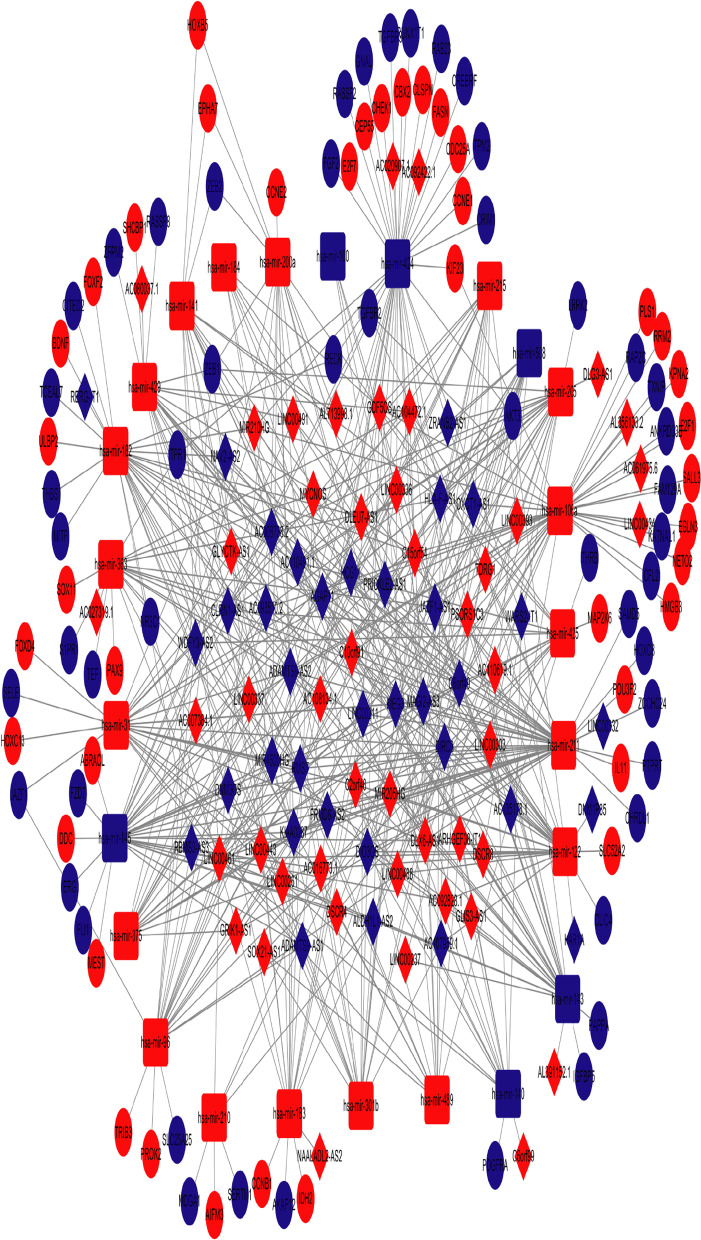
CeRNA network in endometrial carcinoma. The blue nodes represent decreased expression levels, and the red nodes represent increased expression levels. Rectangles represent miRNAs, ellipses represent protein-coding genes, and diamonds represent lncRNAs; gray edges indicate lncRNA-miRNA-mRNA interactions

A total of 2731 differentially expressed mRNAs were obtained, 1566 of which were up-regulated and 1165 were down-regulated. A total of 979 differentially expressed lncRNAs were obtained, 611 of which were up-regulated and 368 were down-regulated. A total of 170 differentially expressed miRNAs were obtained, 127 of which were up-regulated and 43 were down-regulated (Fig. 1, Supplementary Material 1, 2, 3, 4, 5 and 6). miRNA predict the miRNA target genesin miRDB,miRTarBase, TargetScan databases, then the target genes do intersection, get endometrial adenocarcinoma of miRNA- mRNA relations. The mRNA in ceRNA was obtained by intersection of the predicted target gene and the selected differential RNA (Fig. 1d)., The constructed ceRNA network diagram of differentiated RNAs in endometrial adenocarcinoma is shown in Fig. 2, including 90 mRNAs, 81 lncRNAs, and 25 miRNAs.

### WGCNA

**Fig. 3:**
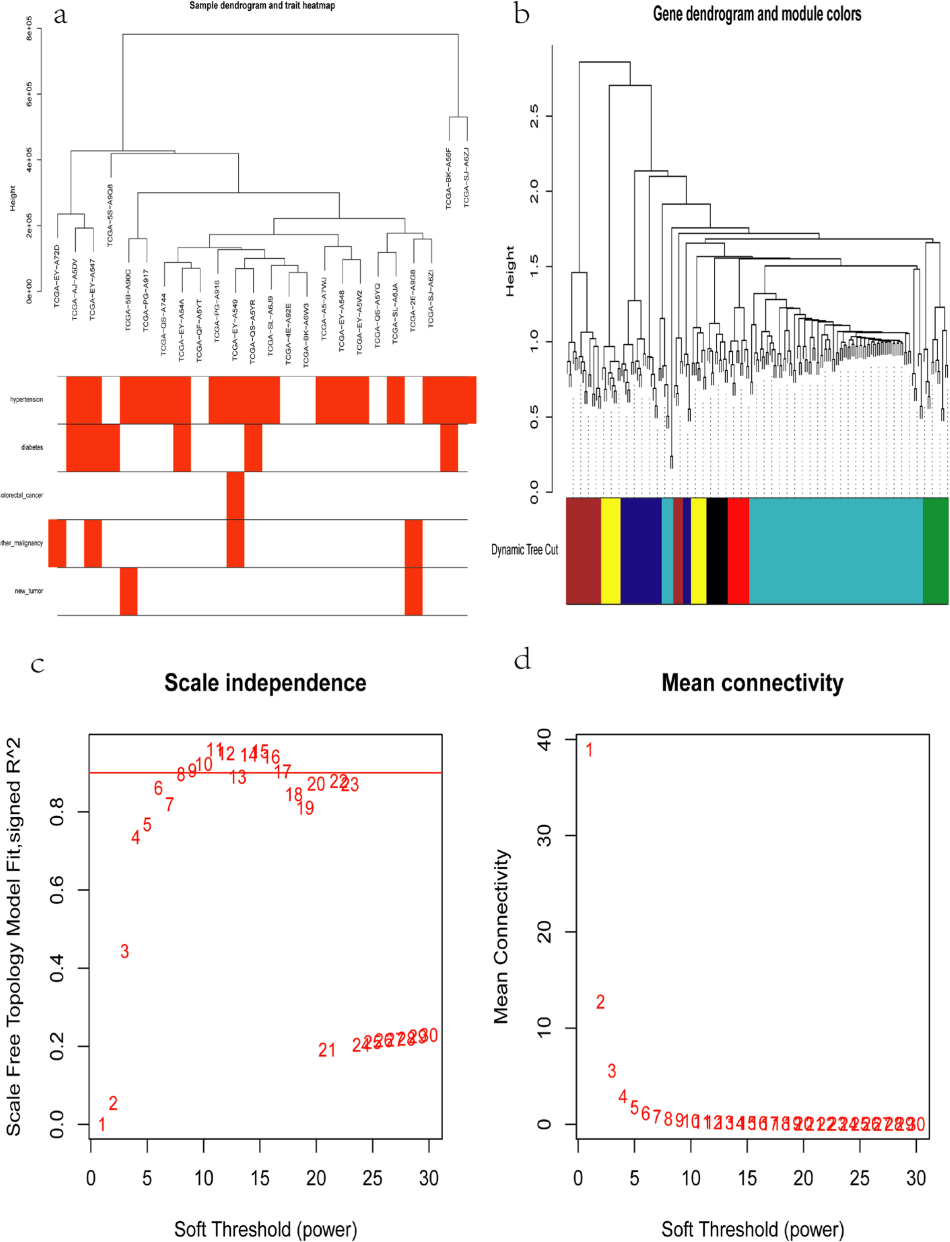
**a** Sample clustering and corresponding heat map of trait data after removing abnormal samples. **b** Cluster dendrogram of genes in 26 TCGA samples. Each branch represents one gene, and every color below represents one co-expression module. **c** Analysis of the scale-free ft. index for various soft-thresholding powers (β). (**d**) Analysis of the mean connectivity for various soft-thresholding powers

**Fig. 4:**
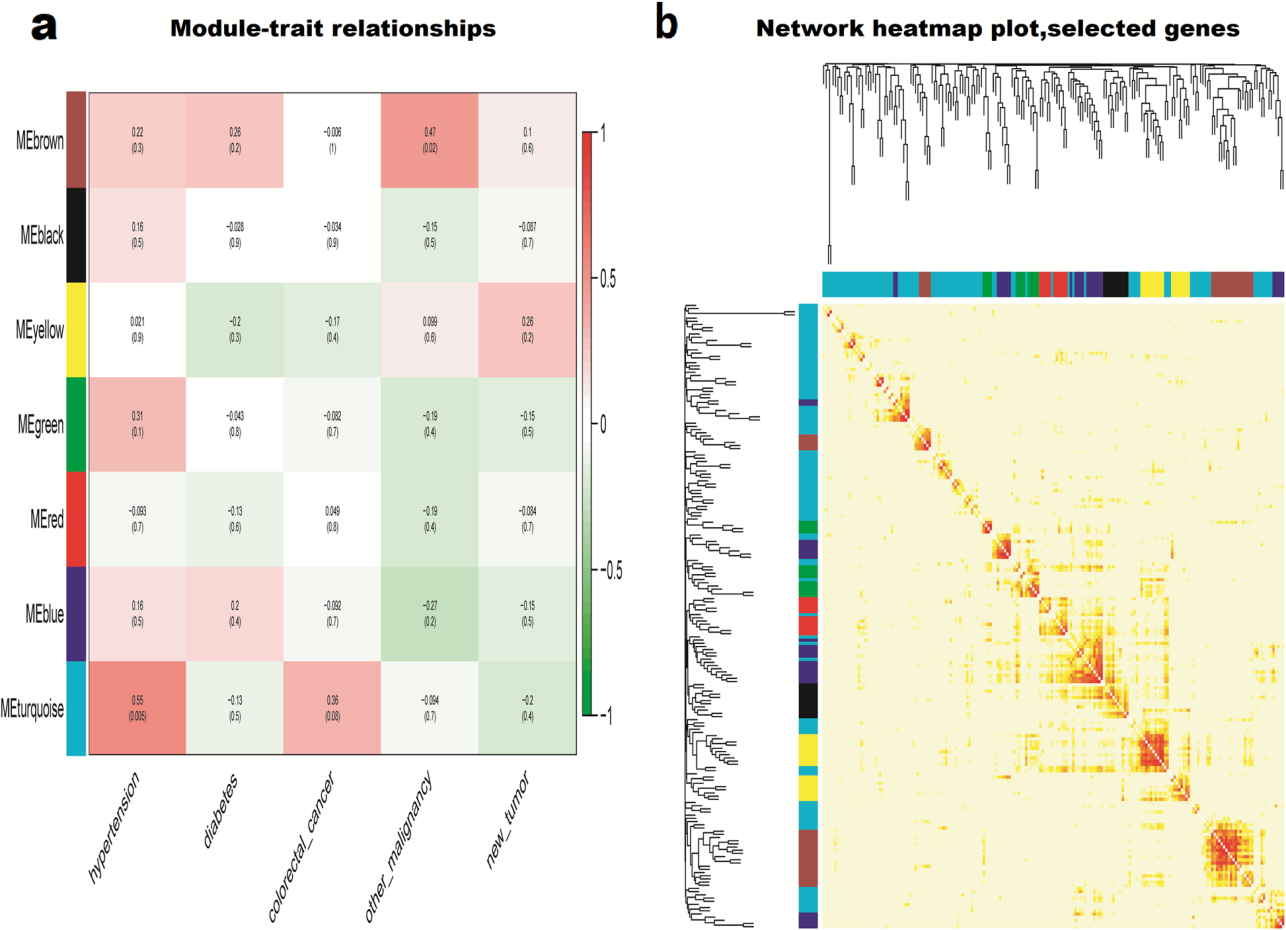
**a** Heatmap of the correlation between module eigengenes and the disease status of endometrial cancer. **b** Interaction relationship analysis of co-expressed genes. Different colors of the horizontal axis and vertical axis represent different modules. The brightness of yellow in the middle represents the degree of connectivity of different modules. There was no significant difference in interactions among different modules, indicating a high-scale independence degree among these modules

**Fig. 5:**
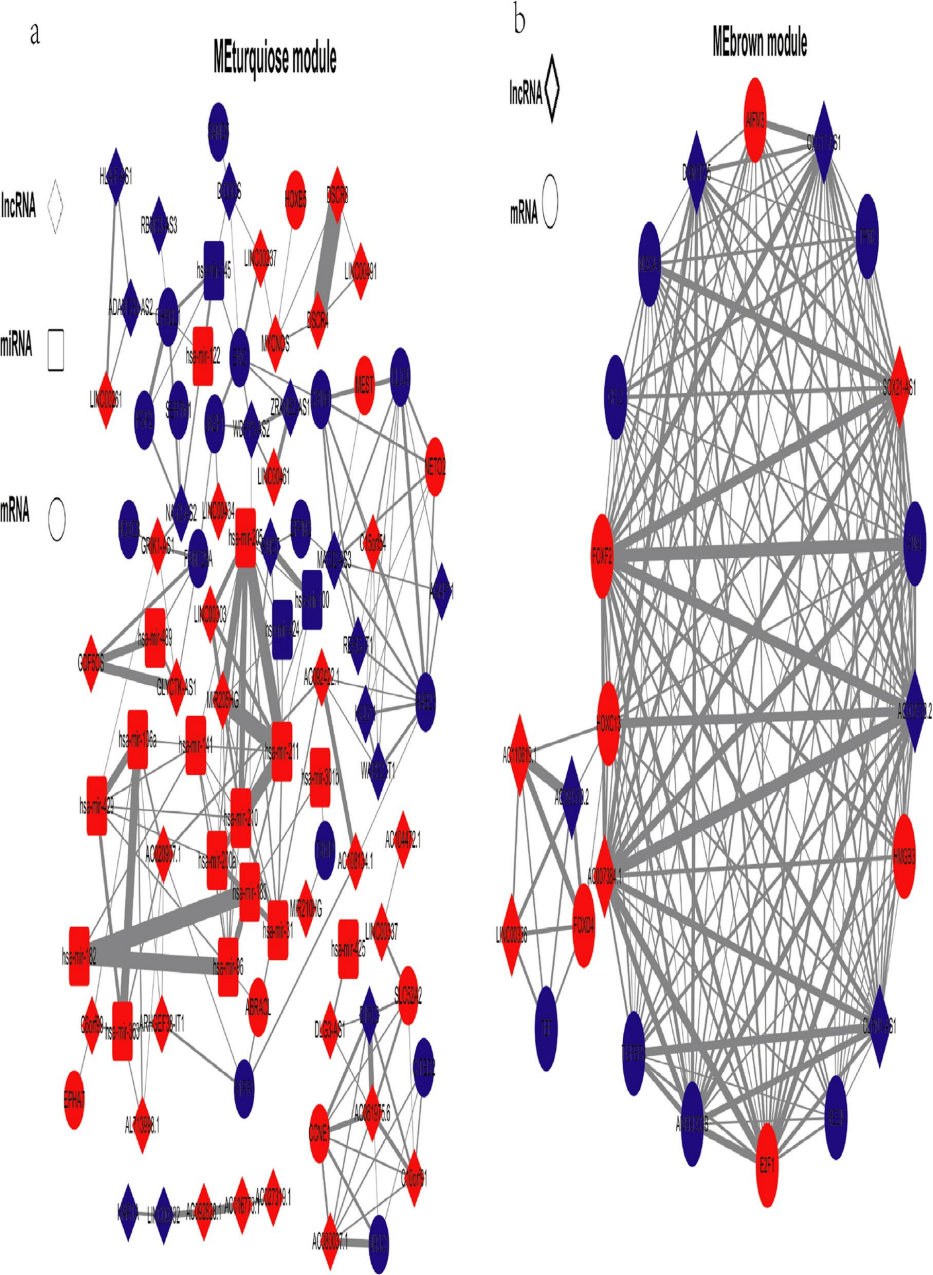
CeRNA network in the MEturquoise and MEbrown modules in endometrial carcinoma. The blue nodes represent decreased expression levels, and the red nodes represent increased expression levels. Rectangles represent miRNAs, ellipses represent protein-coding genes, and diamonds represent lncRNAs; gray edges indicate lncRNA-miRNA-mRNA interactions. The edge width is correlated with the degree of connectivity of the gene

Twenty-seven cancer samples were randomly selected from the TCGA data for further WGCNA analysis of the 196 differentially expressed RNAs in the ceRNA network. The heat map of the correlations between patient characteristics and RNA expression is shown in Fig. 3a. A total of seven modules were constructed: MEbrown, MEblack, MEyellow, MEgreen, MEred, MEblue, and MEturquiose (Fig. 3b). Based on the final scale-free network constructed (Fig. 3c–d), the GeneSignificance(GS) and Module membership (MM) values of the modules were calculated according to combined analysis of modules and trait data along with mining the modules and key genes that play a key role in endometrial adenocarcinoma (Supplementary Material 7). The modules and trait data were found to be significantly correlated, and the heat map of these relationships is shown in Fig. 4a. Overall, this analysis indicated that hypertension was positively correlated with the MEturquiose module (*p* = 0.005), and history of other malignancy was positively correlated with the MEbrown module (*p* = 0.02). The gene clustering tree and heat map of the network are shown in Fig. 4b, and the genes in these two modules were input into Cytoscape (3.7.0) to obtain the weighted ceRNA network graph (Fig. 5).

### GO and KEGG enrichment analysis

**Fig. 6:**
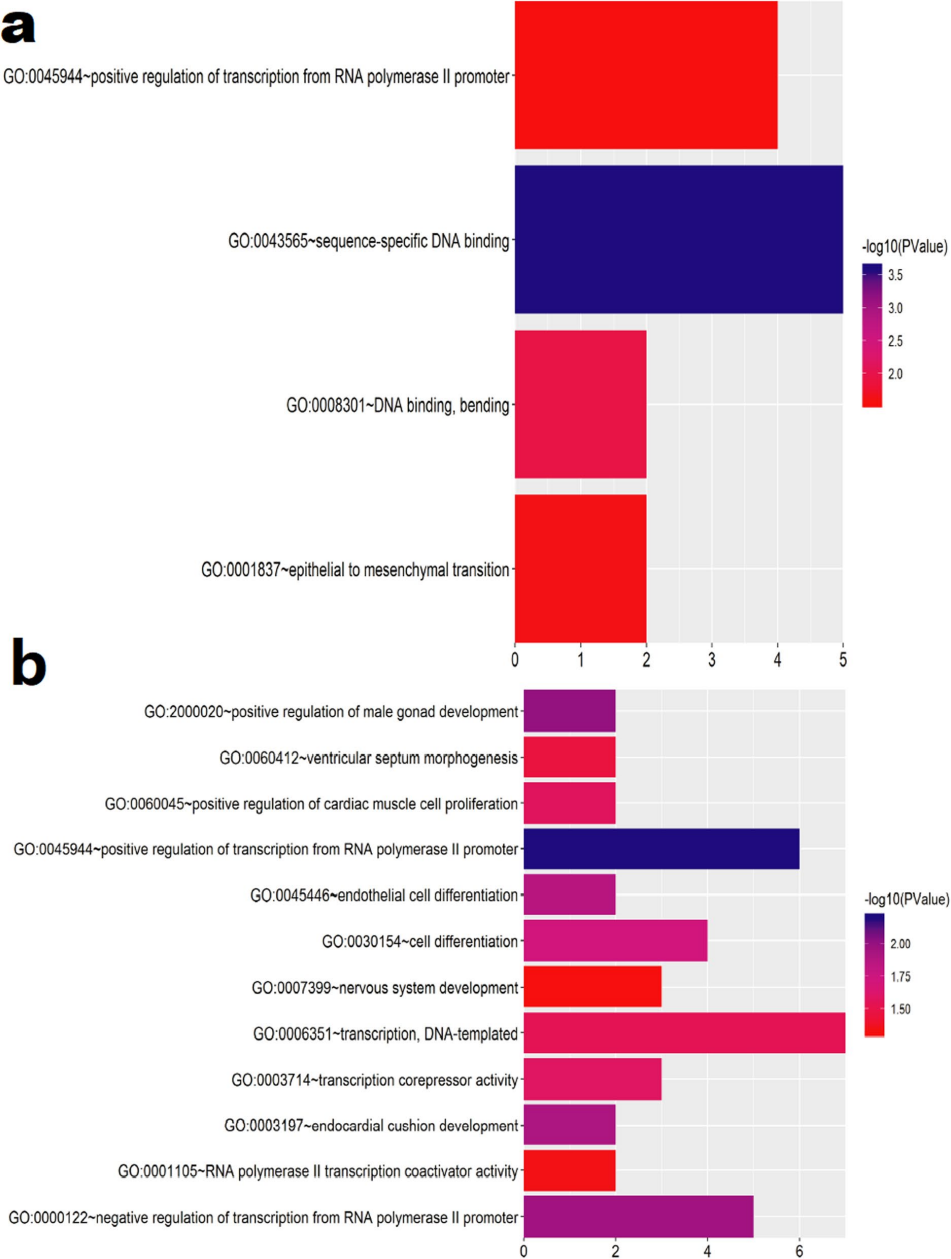
Significantly enriched GO terms of the MEturquiose and MEbrown modules for differentially expressed ceRNAs in endometrial carcinoma (EC). Gene Ontology (GO) enrichment analysis was performed using DAVID. A *P*-value was obtained using a hypergeometric test; *P* < 0.05 was set as the cut-off for significant GO terms. The y-axis shows GO terms and the x-axis presents the counts of differentially expressed ceRNAs in EC-enriched GO terms. The color scale represents the −log false discovery rate (FDR). **a** MEbrown module; **b** MEturquiose module

**Fig. 7:**
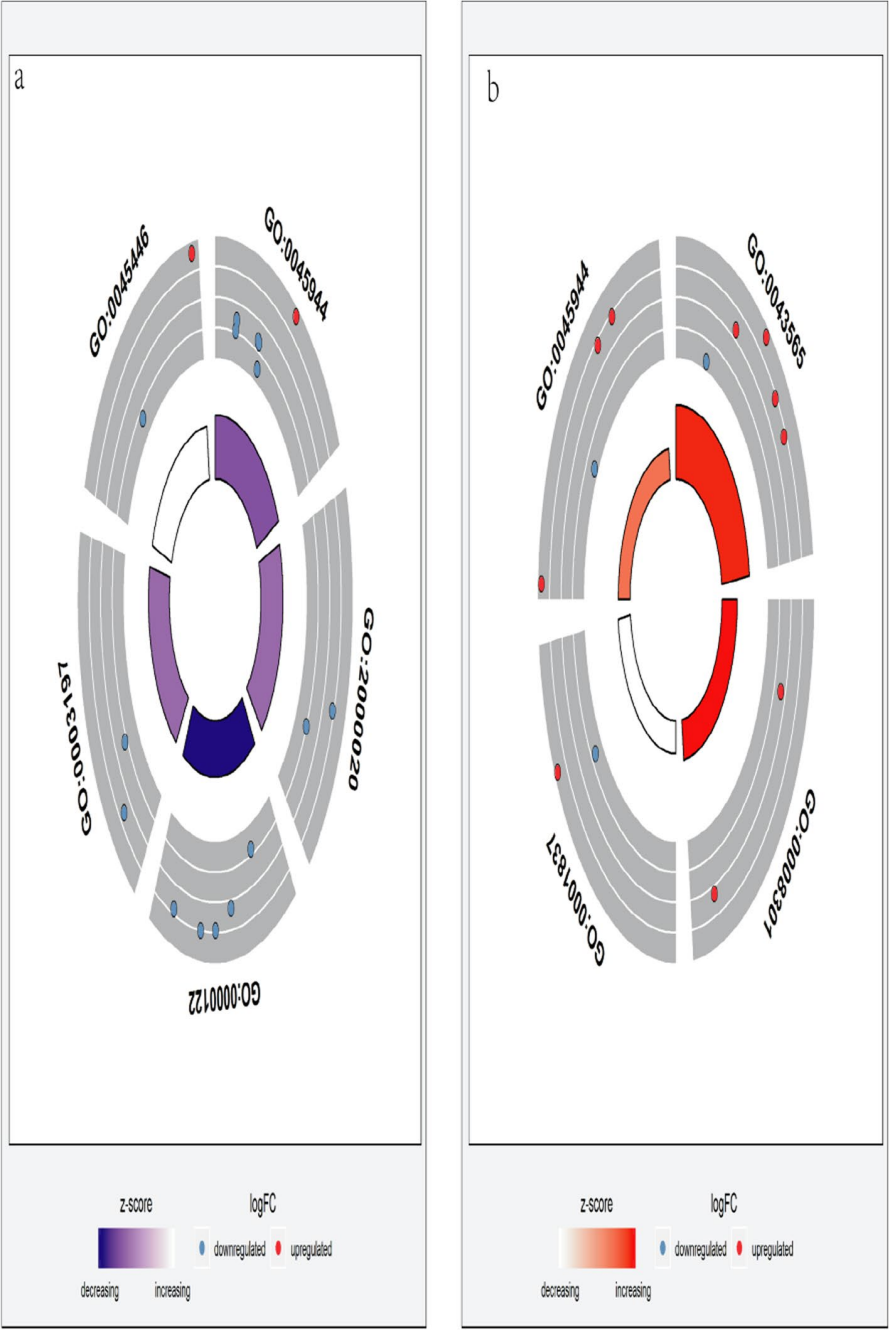
Gene Ontology (GO) enrichment of genes in the (**a**) MEturquiose module and (**b**) MEbrown module. The outer circle shows a scatter plot of the log-fold change for the assigned genes within each distinct GO term. Selection for enrichment resulting in blue and red dots indicates downregulated and upregulation expression, respectively. The inner circle is a bar plot with the height of the bar illustrating the *p-*value of the term and the color representing the *z*-score, which indicates the direction of the fold change of each of the genes within the assigned term and provides a relative scale for enrichment

The GO terms related to these topology modules indicated important functional features. The MEbrown topology module was significantly enriched in four GO terms (Supplementary Material 8): GO 0043565 (*p* = 2.44E-04), closely related to the molecular function of sequence-specific DNA binding; GO 0080301 (*p* = 0.012), closely related to the molecular function of DNA binding/bending; GO 001837 (*p* = 0.024), associated with the biological process of epithelial-to-mesenchymal transition; and GO 0045944 (*p* = 0.029), associated with the biological process of the positive regulation of transcription from RNA polymerase II promoter (Fig. 6a). The MEturquiose module was significantly enriched in 12 GO terms (Fig. 6b Supplementary Material 9). A circle diagram was used to represent the top five GO enrichment terms with the most significant differences, which were all associated with biological process terms: GO 0045944 (*p* = 0.006), associated with positive regulation of transcription from RNA polymerase II promoter; GO 2000020 (*p* = 0.009), associated with positive regulation of male gonad development; GO 0000122 (*p* = 0.011), associated with negative regulation of transcription from RNA polymerase II promoter; GO 0003197 (*p* = 0.012), associated with endocardial cushion development; and GO 0045446 (*p* = 0.014), associated with endothelial cell differentiation (Fig. 7). Pathway enrichment analysis (KOBAS 3.0) of the genes in the MEbrown module showed that *RECK* and *E2F1* were enriched in the miRNA in cancer pathway (*p* < 0.05), with no significant enrichment detected for the other genes. However, there was no significant pathway enrichment for genes in the MEturquiose module (Supplementary Material 10, 11) [21–23].

### Selection and verification of hub genes

**Fig. 8:**
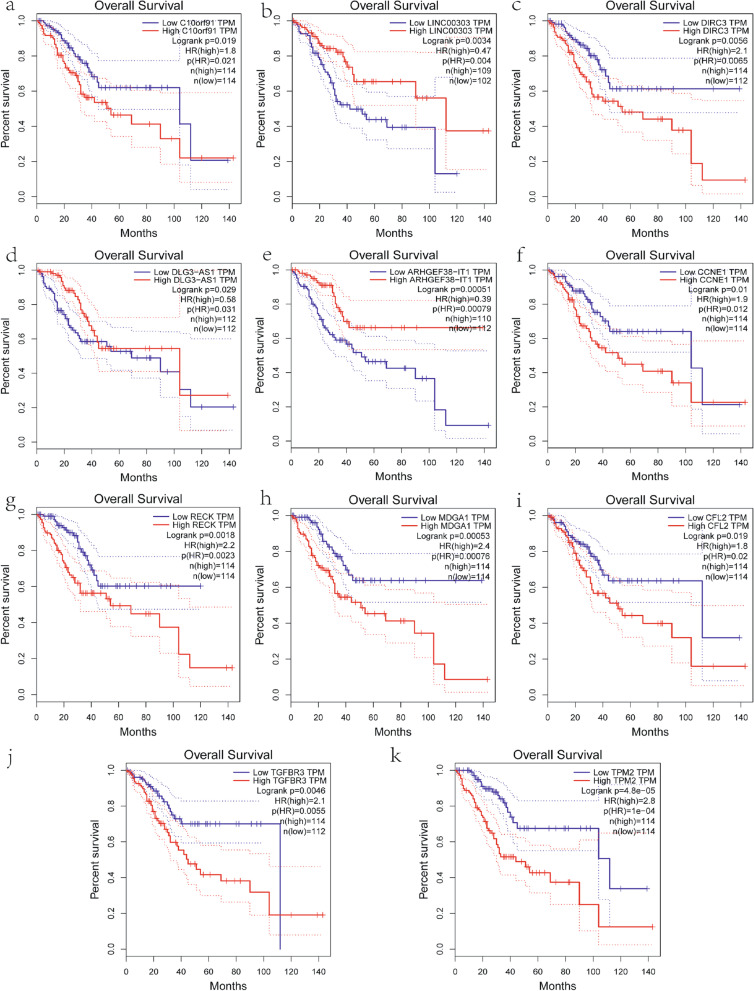
Survival analysis of hub genes. **a**–**f** Six hub genes in the MEturquoise module with the highest node degree and significant results in the survival analysis (*P* < 0.05): *C10orf91, LINC00303, DIRC3, DLG3-AS1, ARHGEF38-IT1*, and *CCNE1*, respectively. **g**–**k** Five hub genes in the MEbrown module with the highest node degree and significant results in the survival analysis (*P* < 0.05): *RECK, MDGA1, CFL2, TGFBR3*, and *TPM2*, respectively

**Fig. 9:**
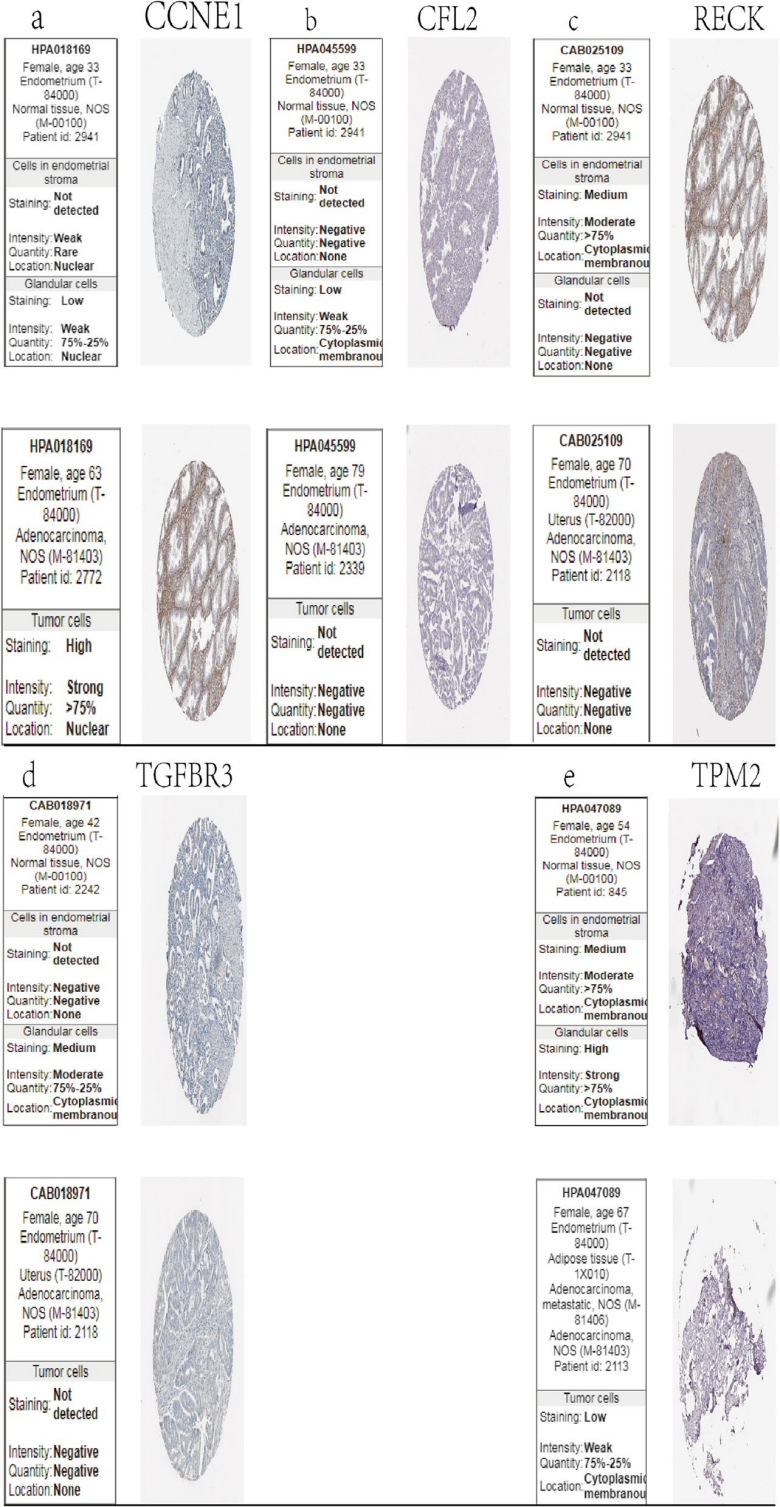
Validation of hub genes at the translational level (**a**–**e**) .Validation of five hub genes in The Human Protein Atlas database (based on immunohistochemistry, IHC)

A total of 33 and nine genes in the MEbrown and MEturquiose module, respectively, showed a nodal degree > 3 and were thus selected as candidate hub genes. GEPIA analysis of these genes (log rank < 0.05) further identified *C10orf91, LINC00303, DIRC3, DLG3-AS1, ARHGEF38-IT1*, and *CCNE1* as hub genes in MEturquoise, and *RECK, MDGA1, CFL2, TGFBR3*, and *TPM2* as hub genes in MEBrown (Fig. 8). The transcriptional levels of these candidate hub genes were verified in The Human Protein Atlas database. There was no significant difference in the protein expression of MDGA1 between normal tissues and endometrial adenocarcinoma tumor tissues. Unfortunately, *C10orf91, LinC00303*, and *DIRC3* are lncRNAs, *DLG3-AS1* is an antisense gene, and *ARHGEF38-IT1* is a sense-intronic lncRNA; therefore, there are no data related to their expression in the Human Protein Atlas (Fig. 9). The 10 hub genes have been listed in Supplementary Material 12.

### Genetic alteration of hub genes

**Fig. 10:**
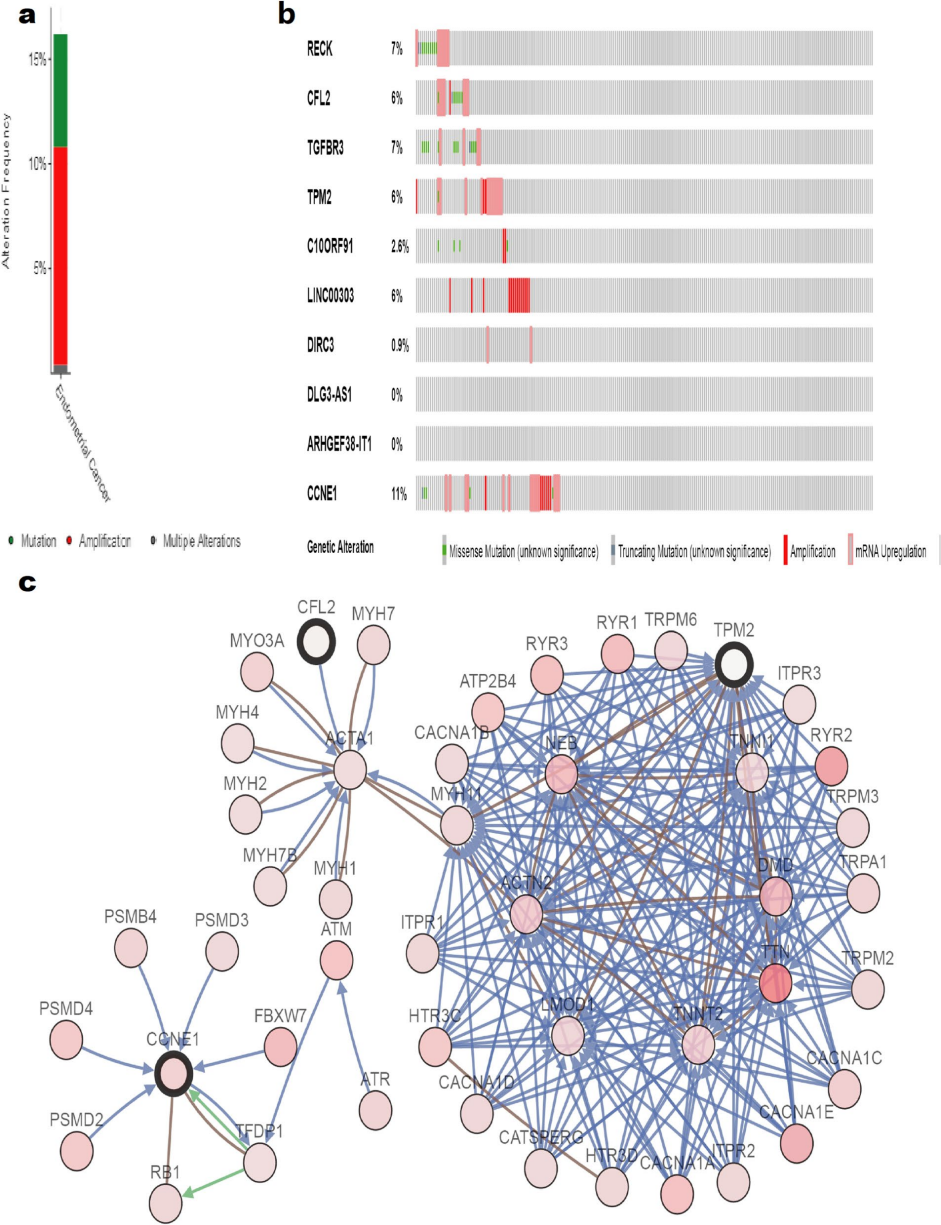
Genetic alterations associated with hub genes in the TCGA endometrial carcinoma (EC) database. **a** Total alteration frequency of 10 hub genes in TCGA EC. **b** Visual summary of the genetic alterations of 10 hub genes in TCGA EC patients. **C** The network contains 60 nodes, including our 10 query genes and the 50 most frequently altered neighbor genes (only three out of the 10 hub genes were correlated with the 50 genes)

**Fig. 11:**
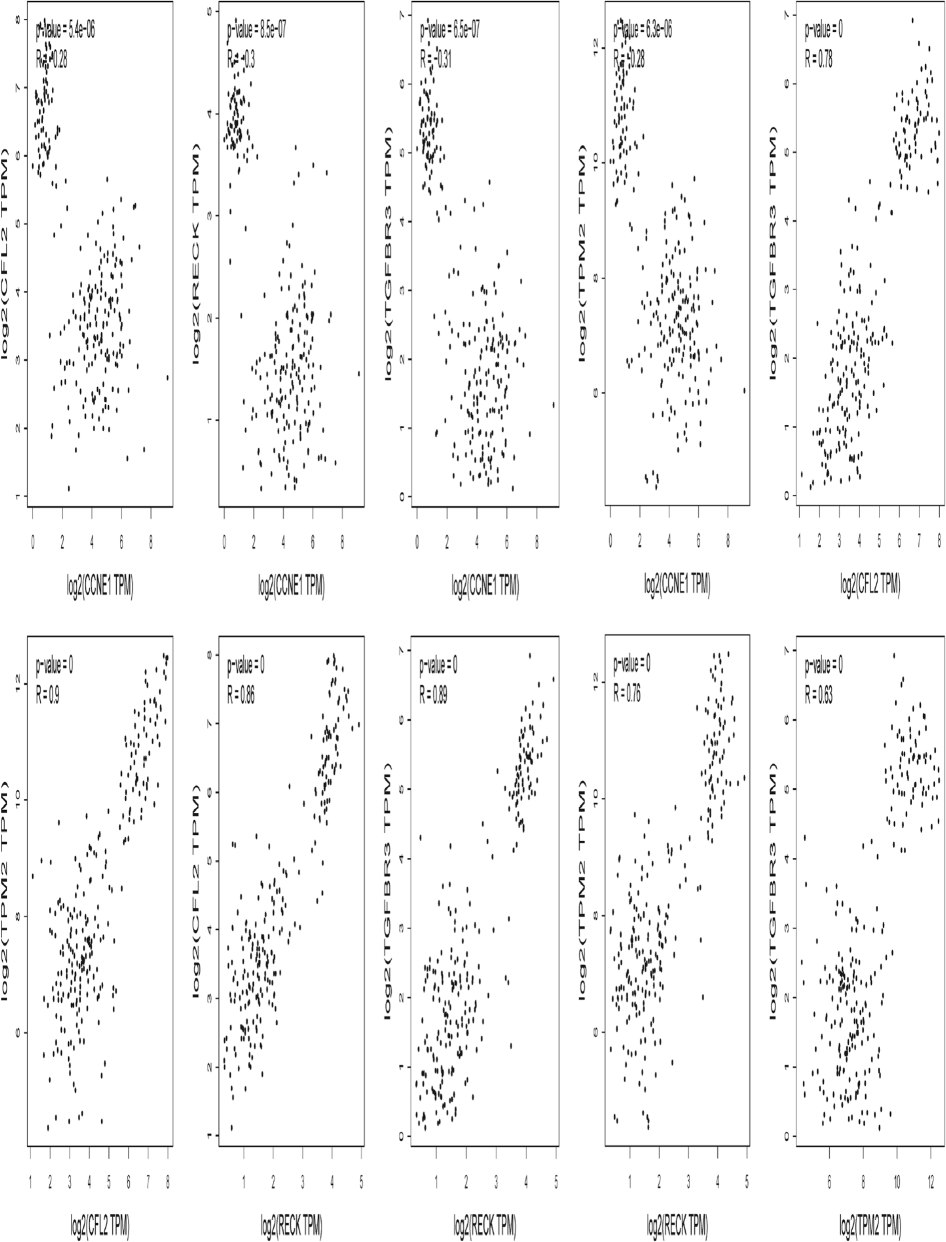
The correlation analysis of the mRNAs *RECK, CFL2, TGFBR3, TPM2*, and *CCNE1* in the GEPIA website

After *MDGA1* was removed, the remaining candidate hub genes (*RECK, CFL2, TGFBR3*, *TPM2,* and *C10ORF91*) affecting the survival rate of endometrial adenocarcinoma in the MEbrown module and MEturquoise module were selected. Based on the oncoPrint map in cBioportal, these 10 hub genes were found to be altered in 73 (31%) of 232 queried patient samples (Fig. 10b), with the greatest degree of alterations detected for *CCNE1, RECK, CFL2, TGFBR3, TPM2*, and *LINC00303,* detected in 11, 7, 6, 7, 6, and 6% of the samples, respectively. The main types of alterations detected were missense mutation, amplification, and mRNA upregulation, although only amplification was found for *LINC00303* alterations (Fig. 10a). Figure 10c demonstrates the relationship of the 10 genes and the other 50 most frequently altered neighboring genes; however, only *CFL2, CCNE1*, and *TPM2* had connections with these 50 genes. There was no known drug targeting these 10 hub genes, indicating promising targets of new cancer drugs. In the MEturquiose module, *C10orf91, LINC00303, DIRC3, DLG3-AS1*, and *ARHGEF38-IT1* are all lncRNAs with no protein expression data available. The correlation analysis of the mRNAs *RECK, CFL2, TGFBR3, TPM2*, and *CCNE1* in the GEPIA website demonstrated a significant negative correlation between *CCNE1* and the other hub genes in the MEbrown module; the other hub genes in the MEbrown module were all significantly positively correlated with each other (Fig. 11).

## Discussion

EC is a common malignant tumor in women, and is one of the three major malignancies of the female reproductive system that seriously affects the quality of life and health of women [24]. Although the prognosis of low-grade EC (grades 1 and 2) is better than that of high-grade EC (grade 3) [25], some patients with low-grade EC still have recurrence and a poor prognosis. Lu et al. [26] recently reported that the incidence of EC was associated with folate-mediated one-carbon metabolism according to a Cox proportional hazards model: with the increase of total folate, natural folate, B6, and B12 intake, there was a significantly increased risk of EC. EC risk has also been associated with greater lifetime number of years of menstruation [27]. Indeed, EC risk is closely related to age, and 90% of cases occur in women older than 45 years [28, 29]. In addition, the risk of EC in patients with diabetes is three times higher than that of the general population, and women with metabolic diseases such as hyperglycemia and obesity should be screened regularly [30, 31]; indeed, obesity, diabetes, and hypertension are together referred to as the “triad of endometrial cancer” [32–34]. Consistently, in the present study, we found a positive correlation between hypertension and the MEturquiose module in the WGCNA. Moreover, GO enrichment analysis showed significant enrichment of GO:0003197, linked to the biological processes of endocardial cushion development.

Soisson et al. [35] conducted a retrospective cohort study, and found that heart disease and other circulatory system conditions were the main causes of death of patients with EC, and patients receiving radiation therapy and/or chemotherapy were found to have a higher risk of heart disease. The results of the present study can provide insight into this relationship between EC and cardiovascular disease from the genetic level. Most lncRNAs are transcribed by RNA polymerase II, which exists in the cytoplasm of eukaryotes, into mRNA primary transcripts. All of the genes in the MEbrown module except for *CCNE1* were lncRNAs, which also explains the significant correlation for GO:0045944 linked to positive and negative regulation of transcription from RNA polymerase II promoter, respectively.

Although there has been no large population-based case-control study on the correlation between EC and other tumors, Schildkraut et al. [36] found a significant association between breast cancer and ovarian epithelial tumor with EC in familial cases. In addition, Liu et al. [37] found that overexpression of syncytin-1 promoted the invasion and metastasis of EC cells by activating epithelial-to-mesenchymal transition-related pathways. Consistently, in this study, the WGCNA confirmed that a history of other malignancies was positively correlated with the MEbrown module. Further, GO analysis of the MEbrown module showed significant enrichment of GO:001837, related to epithelial-to-mesenchymal transition, a well-known important process of tumor metastasis. Moreover, the KEGG analysis showed that *RECK* and *E2FI* in the MEbrown module are enriched in the pathways related to miRNA in cancer. *RECK* is regulated by miR-21 and is involved in the invasion and metastasis of glioblastoma, and has also been shown to affect the survival rate of patients with this type of tumor. *E2F1* is regulated by miR-20a, miR-106b, and miR-330 in prostate cancer and affects cell epigenetic changes related to cell growth (resistance to apoptosis). The discovery of the MEbrown module provides a molecular basis for studying the relationship between EC and tumorigenesis processes of other tumor types at the genetic level. Based on our results, we speculate that factors such as *RECK* and *E2F1* in the MEbrown module directly affect the invasion and metastasis of EC through gene regulation, resulting in the occurrence of other malignant tumors, activation of proto-oncogenes in other parts through exosomes, or promotion of the metastasis of EC to other tissues through the tumor microenvironment.

Zhou et al. [38] experimentally confirmed that large intergenic non-coding ribonucleic acid-RoR is a ceRNA and acts as an miR-145 “sponge” to inhibit the mediation of the differentiation of stem cells by miR-145. Vallone et al. [39] reviewed the relationship between non-coding RNAs and oncogenesis (lung cancer, bladder cancer, kidney cancer, and melanoma) based on 3 years of PubMed records. To date, very few ceRNA network studies have been performed with regards to EC. In the present study, correlation analysis showed that *CCNE1* in the MEturquiose module was significantly negatively correlated with all of the hub genes in the MEbrown module, while all of the hub genes in the MEbrown module were significantly positively correlated, indicating that the two modules are not only modules with respect to topology but also with respect to function, and the existence of two modules is reasonable. In the ceRNA expression network of DEGs, we also found that both *CCNE1* and *TPM2* are only directly regulated by mir-424, and are negatively regulated by each other in mir-424-centered ceRNA; however, they belong to two modules, indicating that the two modules do not exist in isolation, but rather have a certain genetic correlation. *CFL2* is only directly regulated by mir-106a in the ceRNA expression network of DEGs. Linear regulation of *TPM2* and *CFL2* may be directly mediated by mir-106a/oxct1-as1/mir-424 or through the complex ceRNA network between mir-106a and mir-424. This finding provides a new strategy for investigating the mechanism of EC.

Current treatment for EC largely depends on chemotherapy regimens and hormone-based therapy in combination with surgery and radiotherapy [40]. Traditional chemotherapy drugs include platinum-based anti-neoplastics, taxanes, nucleoside analogues, immune modulators, fibroblast growth factor receptor and tyrosine kinase inhibitors, small-molecule mTOR inhibitors, and drugs that trigger cell cycle arrest in the G1 phase [41–43]. With the discovery of biomarkers of EC, some targeted therapies such as bevacizumab have shown remarkable anticancer effects [44]. By screening the MEturquoise and MEbrown modules, we identified *RECK, CFL2, TGFBR3, TPM2, C10ORF91, LINC00303, DIRC3, DLG3-AS1, ARHGEF38-IT1*, and *CCNE1* as hub genes. The discovery of these two modules provides a basis for further research on the mechanism of the occurrence and development of EC. Moreover, the newly identified 10 hub genes might serve as prognostic biomarkers and therapeutic targets in the future. In addition, no known anti-tumor targets of the 10 hub genes were predicted in cBioportal, which could provide new ideas for the development of targeted drugs in EC.

## Conclusions

We analyzed the differentially expressed RNAs of endometrial adenocarcinoma based on TCGA data, and established a ceRNA network. The identified ceRNAs may play a critical role in the progression and metastasis of EC, and are thus candidate therapeutic targets and potential prognostic biomarkers. The two modules identified provided a valuable reference that will advance studies into understanding the mechanisms of tumorigenesis in EC.

## Supplementary Information

**Additional file 1.** Up-mRNA. Up regulation of mRNA.

**Additional file 2.** Down-mRNA. Down regulation of mRNA.

**Additional file 3.** Up-lncRNA. Up reglation of lncRNA.

**Additional file 4.** Down-lncRNA. Down regulation of lncRNA.

**Additional file 5.** Up-miRNA. Up regulation of miRNA.

**Additional file 6.** Down-miRNA. Down regulation miRNA.

**Additional file 7.** GS&MM. The value of GS and MM in each module.

**Additional file 8.** MEbrown DAVID. DAVID enrichment of MEbrown.

**Additional file 9.** MEturquiose DAVID. DAVID enrichment of MEturquiose.

**Additional file 10.** KEGG pathway. KEGG pathway enrichment of MEbrown module.

**Additional file 11.** KEGG pathway. KEGG pathway enrichment of MEbrown module.

**Additional file 12.** 10 hub genes. The location and function for 10 hub genes.

## Data Availability

The datasets analyzed during the current study are available in the TCGA repository (Data generated in the study can be accessed from theadditional files).

## Abbreviations

EC: Endometrial carcinoma
TCGA: The Cancer Genome Atlas
MiRNA: MicroRNA
lncRNA: Long non-coding RNA
ceRNA: Competing endogenous RNA
WGCNA: Weighted gene co-expression network analysis
GO: Gene Ontology
KEGG: Kyoto Encyclopedia of Genes and Genomes
DAVID: Database for Annotation, Visualization and Integrated Discovery
MRE: MicroRNA response element
DEG: Differentially expressed gene
