# Supplementary figures and images for: Construction and comprehensive analysis of the competing endogenous RNA network in endometrial adenocarcinoma
Source: BMC Genom Data. 2022 Feb 6;23:10. doi: 10.1186/s12863-022-01028-y (PMC8818217; doi:10.1186/s12863-022-01028-y)

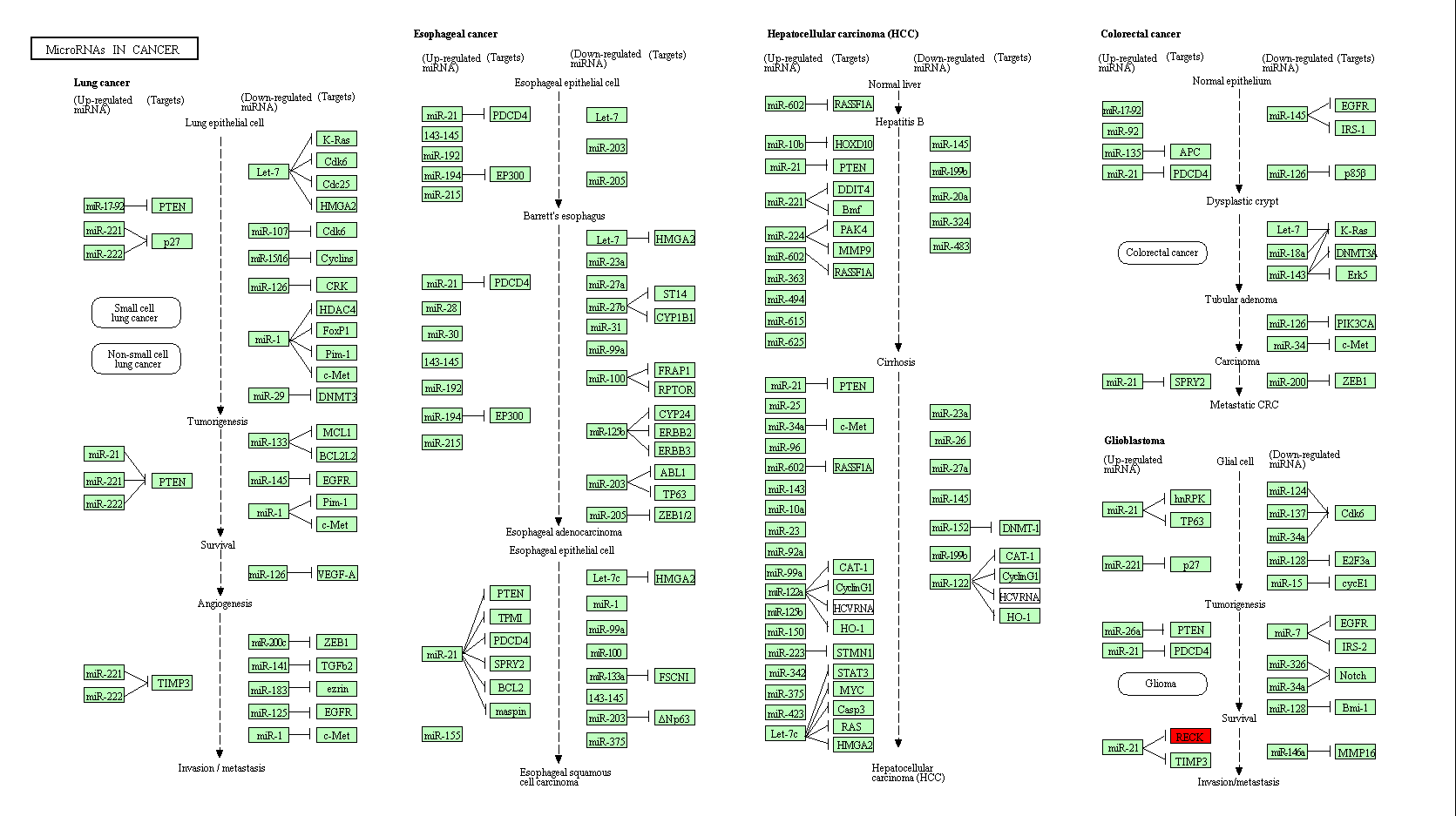

Supplement: Supplementary file 10 — Additional file 10. KEGG pathway. KEGG pathway enrichment of MEbrown module. [file 12863_2022_1028_MOESM10_ESM.png]

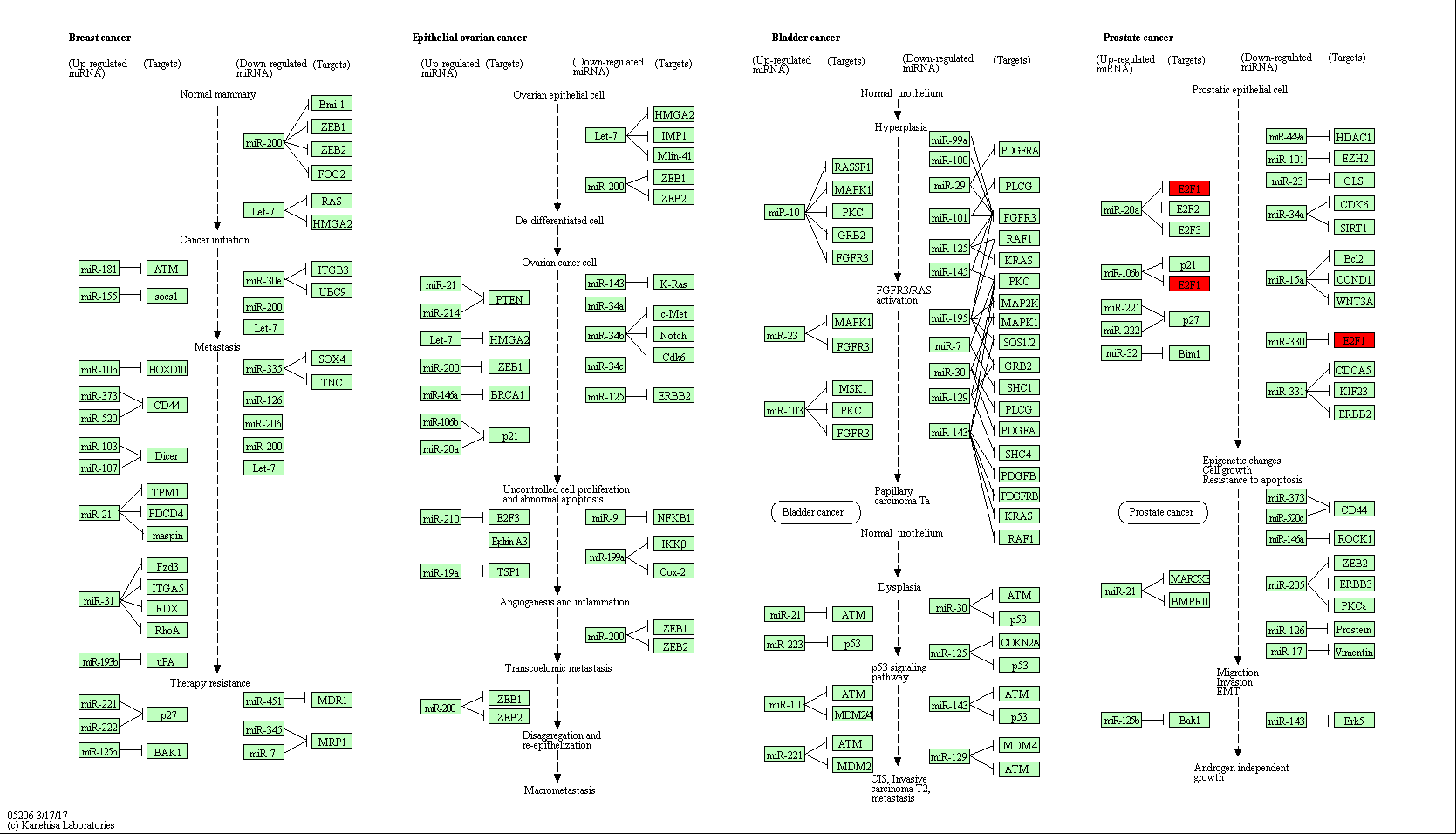

Supplement: Supplementary file 11 — Additional file 11. KEGG pathway. KEGG pathway enrichment of MEbrown module. [file 12863_2022_1028_MOESM11_ESM.png]
